# Emerging Paradigms in Molecular Pharmacology: Disease Mechanisms, RNA Therapeutics, and AI-Driven Drug Design

**DOI:** 10.3390/cimb48100969

**Published:** 2026-09-22

**Authors:** Igor F. Tsigelny

**Affiliations:** Department of Neurosciences, University of California San Diego, La Jolla, CA 92093, USA; itsigeln@ucsd.edu

Stepping into the role of Section Editor-in-Chief for Molecular Pharmacology at *Current Issues in Molecular Biology* (*CIMB*) comes at a pivotal moment for the discipline. Since MDPI assumed publication of the journal in 2021, this section has evolved from a repository for classical descriptive pharmacology into a highly interdisciplinary platform. The modern drug discovery pipeline no longer operates in isolated silos; it now demands a continuous, seamless feedback loop between deep disease pathology, structural biochemistry, and translational therapeutic models. My core objective moving forward is to ensure our publications reflect this integration, prioritizing papers that move past simple phenotypic observations to provide genuine mechanistic depth that advances personalized medicine.

The thematic trajectory of the section over the last five years mirrors these broader technological shifts in biomedical science. In 2021 and 2022, our volumes were heavily anchored in preclinical validation. These papers established crucial baselines by pairing in vitro screens with targeted in vivo rodent models to map cellular signaling pathways and understand the fundamental molecular mechanisms of diseases. 

By 2023 and 2024, however, the section experienced a clear structural shift driven by high-throughput methodologies and genomic insights. A major catalyst was the explosion of research detailing the role of non-coding RNA (ncRNA) in disease mechanisms. Authors transitioned from viewing microRNAs (miRNAs), long non-coding RNAs (lncRNAs), and circular RNAs as genomic noise to recognizing them as vital regulatory nodes and primary therapeutic targets. Concurrently, advanced computational profiling transformed early-stage drug design, moving virtual screening from a secondary afterthought to a primary pipeline filter. 

Our 2025 and 2026 issues reflect a sharp focus on precision oncology, targeted delivery, and advanced computational intelligence. We are seeing a significant volume of work focused on elucidating how pathologically altered cellular systems bypass current chemotherapeutic agents. Crucially, the section is now covering the exciting field of AI-based selection of drug combinations. By leveraging machine learning algorithms and knowledge graphs, researchers identify synergistic multi-target combinations capable of overcoming complex drug resistance networks within individual patient profiles. 

As we look toward future volumes, the editorial team will specifically seek out manuscripts that push these technical and conceptual boundaries. To maintain high academic rigor and maximize citation impact, we are establishing clear priorities for future submissions:

- **Deciphering Non-Coding RNA and Disease Pathophysiology:** We highly encourage submissions that explore the precise molecular mechanisms by which ncRNAs drive disease progression or drug resistance. Papers must bridge the gap between target discovery and innovative methods for treating them, such as chemically modified RNA therapeutics, antisense oligonucleotides, or nanocarrier-mediated delivery systems.
- **Structure-Guided Drug Design:** We seek papers showcasing next-generation small-molecule or biologic design. Studies should leverage advanced computational modeling, molecular dynamics simulations, or biophysical kinetics to demonstrate precise target engagement and optimized pharmacological properties.
- **AI-Enhanced Systems Pharmacology and Combination Therapies:** We welcome computational frameworks that utilize artificial intelligence to design, predict, and validate multi-drug regimens. Submissions must demonstrate how AI algorithms map complex biological networks to select optimal drug combinations, accompanied by robust benchside validation.
- **Translational Personalized Medicine:** We prioritize research that advances patient stratification and endotype-specific targeting. Rather than reporting blanket therapeutic effects, studies should investigate how genetic or molecular biomarkers dictate individual drug response, tolerance, and efficacy.

The impact of this section rests entirely on the rigor of our contributors, reviewers, and Editorial Board Members. I welcome your high-quality original research, focused updates, and competitive Special Issue proposals as we work together to shape the future of molecular therapeutics. 

